# The catalytic power of magnesium chelatase: a benchmark for the AAA ^+^ ATPases

**DOI:** 10.1002/1873-3468.12214

**Published:** 2016-06-02

**Authors:** Nathan B. P. Adams, Amanda A. Brindley, C. Neil Hunter, James D. Reid

**Affiliations:** ^1^ Department of Molecular Biology and BiotechnologyUniversity of SheffieldUK; ^2^ Department of ChemistryUniversity of SheffieldUK

**Keywords:** ATP hydrolysis, ATPases associated with various cellular activities (AAA) magnesium protoporphyrin IX, chelatase, chlorophyll biosynthesis, Gun4

## Abstract

In the first committed reaction of chlorophyll biosynthesis, magnesium chelatase couples ATP hydrolysis to the thermodynamically unfavorable Mg^2+^ insertion into protoporphyrin IX (ΔG°′ of circa 25–33 kJ·mol^−1^). We explored the thermodynamic constraints on magnesium chelatase and demonstrate the effect of nucleotide hydrolysis on both the reaction kinetics and thermodynamics. The enzyme produces a significant rate enhancement (*k*_cat_/*k*_uncat_ of 400 × 10^6^ m) and a catalytic rate enhancement, $k_{\mathrm{cat}}/K_{m}^{\mathrm{DIX}}K_{0.5}^{\mathrm{Mg}}k_{\mathrm{uncat}}$, of 30 × 10^15^ m ^−1^, increasing to 300 × 10^15^ m ^−1^ with the activator protein Gun4. This is the first demonstration of the thermodynamic benefit of ATP hydrolysis in the AAA ^+^ family.

## Abbreviations

**D_IX_**, deuteroporphyrin IX

**MgD_IX_**, magnesium deuteroporphyrin IX

**Figure 1:**
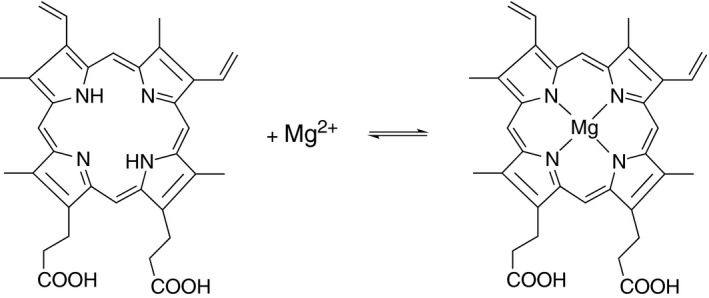
The insertion of Mg^2+^ into the ring of deuteroporphyrin.

Magnesium chelatase (EC 6.6.1.1) catalyzes the first committed reaction of chlorophyll biosynthesis—the insertion of Mg^2+^ into the protoporphyrin IX macrocycle. The chelatase reaction (Fig. 1) is thermodynamically unfavorable with a ΔG°′ of circa 25–33 kJ·mol^−1^ and is coupled to the hydrolysis of MgATP^2−^ 1. Magnesium chelatase from *Synechocystis* PCC6803 has an ATPase stoichiometry of 15 while the *Rhodobacter capsulatus* enzyme has a stoichiometry of 40 1, 2.

Magnesium chelatase is formed from three types of subunit; only one subunit, ChlI, has a confirmed ATPase activity, although the ChlD subunit is also a member of the AAA^+^ superfamily of ATPases 3, 4 and allosterically regulates the chelatase 5, 6, 7. This superfamily couples ATP hydrolysis to a wide variety of intracellular reactions, using the free energy of nucleotide hydrolysis to drive cellular processes that often, but not exclusively, involve macromolecular remodeling 8, 9. These ATP hydrolysis and remodeling activities are indirectly linked through protein conformational changes; in general, AAA^+^ enzymes do not phosphorylate their substrates.

Many AAA^+^ enzymes have high ATPase stoichiometries, but ATP hydrolysis is not always required to provide a thermodynamic driving force 1. In order to understand the significance of nucleotide hydrolysis in these reactions, we need to understand the thermodynamic constraints and the effects of nucleotide hydrolysis on both the reaction kinetics and thermodynamics. We show here that ATP hydrolysis catalyzed by magnesium chelatase shifts the ratio of products to substrates ([MgD_IX_]/[Mg^2+^][D_IX_]) for the metal ion chelation reaction from the equilibrium ratio of 10^−6^ to circa 500 at low porphyrin concentrations. The chelatase can only maintain this ratio in the presence of ATP, and once nucleotide is consumed the ratio decays to the equilibrium position. Additionally, we have measured the first‐order rate constant for magnesium porphyrin dechelation. Using the equilibrium constant and assuming that the chelation process can be described by a second‐order rate constant, we show that the enzyme produces a significant rate enhancement, in the presence of ATP, with *k*_cat_/*k*_uncat_ of 400 × 10^6^ m and a catalytic rate enhancement, $k_{\mathrm{cat}}/K_{m}^{\mathrm{DIX}}K_{0.5}^{\mathrm{Mg}}k_{\mathrm{uncat}}$, of 30 × 10^15^ m ^−1^ corresponding to a transition state disassociation constant (*K*_TX_) in the attomolar range. Taken together, our results provide the first clear view of the catalytic power of a member of the AAA^+^ enzyme superfamily, magnesium chelatase.

## Methods and materials

### Materials

Porphyrins were supplied by Inochem (Carnforth, UK). All other chemicals were purchased from Sigma Aldrich unless otherwise stated.

### Purification of chelatase subunits

The GUN4 gene from *Synechocystis* PCC6803 was subcloned from pGEX‐4T‐1SYNGun4 10 using primers ABGun4SYNNdeI TCCATATGTCTGATAATTTGACC and ABGun4SYNBamHI TCGGATCCTTACCAACCGTATTGGGACC. The PCR product was digested with *Nde*I and *Bam*HI and ligated into pET14b. The expression vectors pET9a‐ChlI, pET9a‐His_6_ChlD, and pET9a‐His_6_ChlH 11 were used to produce recombinant protein. ChlI and His_6_‐ChlH were overexpressed in *E. coli* Rosetta2(DE3) pLysS using 500 mL of the auto‐inducing medium ZYM‐5052 12 at 25 °C for 20 h. His_6_‐ChlD and His_6_‐Gun4 were overexpressed in Rosetta2(DE3) pLysS, grown in LB medium at 37 °C and induced with 0.4 mm IPTG at an absorbance (600 nm) of 0.8–1.0. The temperature was then lowered to 18 °C and cells harvested after 15 h. ChlI, His_6_‐ChlH and His_6_‐ChlD were then purified essentially as described previously 1, 10, 11. His_6_‐Gun4 was purified by metal affinity chromatography and exchanged into 50 mm Tricine pH 7.9, 200 mm NaCl, 300 mm glycerol using a PD10 column (GE Biosciences, Little Chalfont, Bucks, UK), prior to magnesium chelatase assays.

### Assays of enzyme activity

Chelatase assays were performed at 34 °C, *I* = 0.1 (KCl) and pH 7.7 in 50 mm MOPS/KOH, 300 mm glycerol, 1 mm DTT. The reaction was monitored observing product formation using a FluoStar Omega Plate Reader (BMG Labtech, Aylesbury, Bucks, UK) in fluorescence mode, *λ*_ex_ = 420 ± 10 nm, *λ*_em_ = 580 ± 10 nm, as previously described 1.

## Results

### Steady‐state product formation

**Figure 2:**
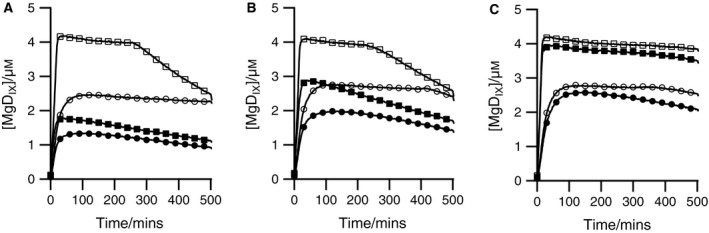
Progress curves showing the characteristic rise and fall of MgD_IX_ during a magnesium chelatase (0.1 μm ChlD, 0.1 μm ChlI, and 0.4 μm ChlH) catalyzed reaction in the presence (open markers) or absence (closed markers) of an ATP‐regenerating system with (squares) or without (circles) 0.4 μm Gun4. All reactions were performed at 34 °C, 50 mm MOPS, 300 mm glycerol, *I* 0.1, pH 7.9, 1 mm DTT and contained 8 μm D_IX_, 10 mm Mg^2+^ and (A) 0.5 mm MgATP ^2−^, (B) 1 mm MgATP ^2−^ or (C) 5 mm MgATP^2−^.

Magnesium chelatase catalyzes the insertion of a Mg^2+^ ion into protoporphyrin (Fig. 1). This reaction shows an unusual but characteristic rise and fall in product concentration (Fig. 2). At the maximum product concentration, the rate of product formation equals the rate of loss so a steady state (*d*[P]/dt ≈ 0) is observed. This steady state is short‐lived unless an ATP‐regenerating system is added, whereupon magnesium chelatase can maintain the steady state for 7 or 8 h (Fig. 2). The steady‐state concentration of MgD_IX_ increases with an ATP‐regenerating system, but only at low initial concentrations of MgATP^2−^, suggesting that the low steady‐state concentrations of MgD_IX_ are due to nucleotide depletion. The activating protein Gun4 provides a small, but consistent increase in the steady‐state ratio of MgD_IX_ to D_IX_ obtained.

**Figure 3:**
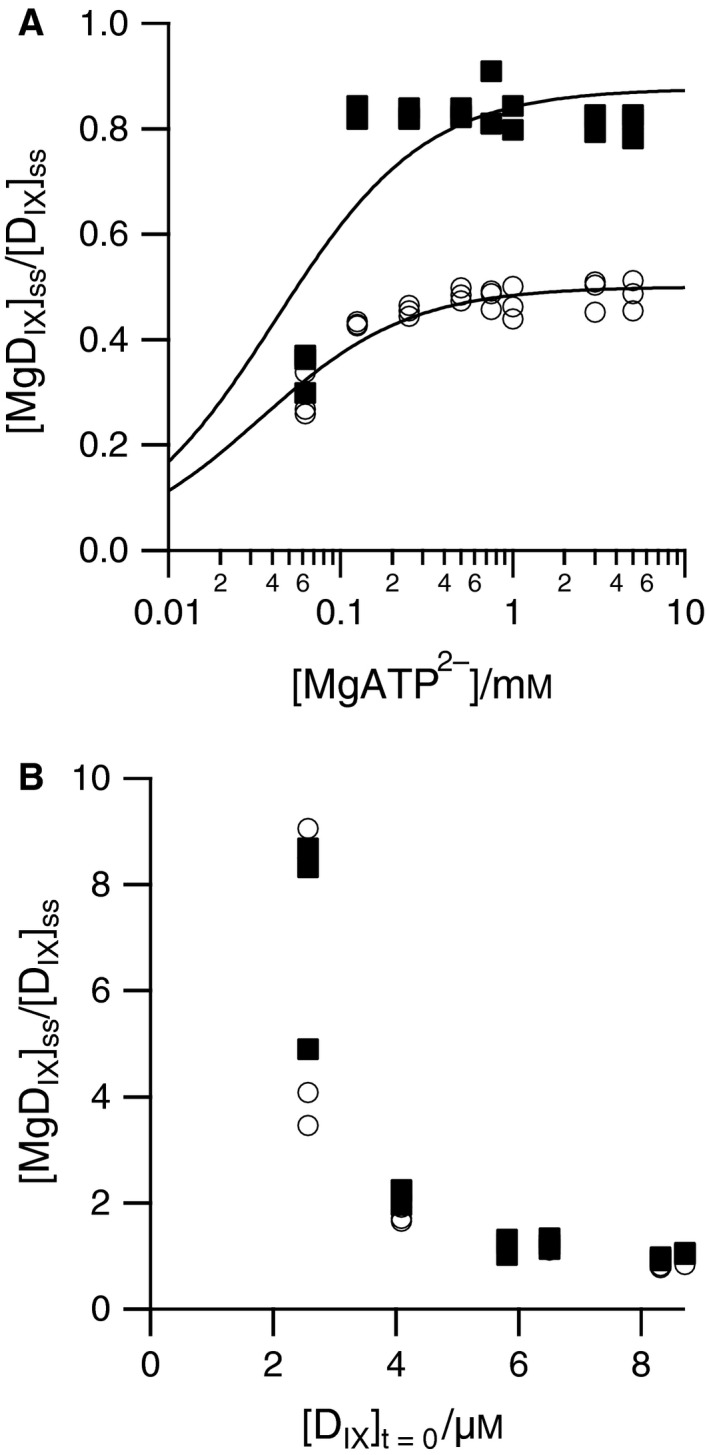
Steady‐state ratios of product to substrate achieved by magnesium chelatase (0.1 μm ChlD, 0.1 μm ChlI, and 0.4 μm ChlH) with (squares) or without (circles) 0.4 μm Gun4 at 34 °C, 50 mm MOPS, 300 mm glycerol*, I* 0.1, pH 7.9, 1 mm DTT 10 mm Mg^2+^, ATP‐regenerating system (2 mm PEP, 2 U·mL^−1^ PK) and (A) varying MgATP ^2−^ at 8 μm D_IX_, (B) varying D_IX_ at 5 mm MgATP ^2−^. The lines in (A) are empirical.

The steady‐state ratio, [MgD_IX_]/[D_IX_]_ss_, depends on the initial concentration of both MgATP^2−^ (Fig. 3A) and porphyrin (Fig. 3B). At high concentrations of MgATP^2−^ this steady‐state ratio tends to 0.5 and the activating protein, Gun4, shifts this ratio to 0.8; these ratios have essentially been achieved at circa 150 μm MgATP^2−^. Low initial concentrations of porphyrin allow a more complete conversion of porphyrin substrate to product (Fig. 3B). At 2.5 μm total porphyrin, the chelatase reaches a steady product:substrate state ratio of around 4 and Gun4 shifts this ratio to circa 8. Increasing total porphyrin concentration reduces the ratio achieved by the enzyme. As these results were obtained at 10 mm free Mg^2+^, they correspond to [MgD_IX_]/[D_IX_][Mg^2+^] ratios of circa 400–800 at low porphyrin concentrations and circa 100 at high porphyrin concentrations.

### Rate of the uncatalyzed dechelation of Mg‐deuteroporphyrin

**Figure 4:**
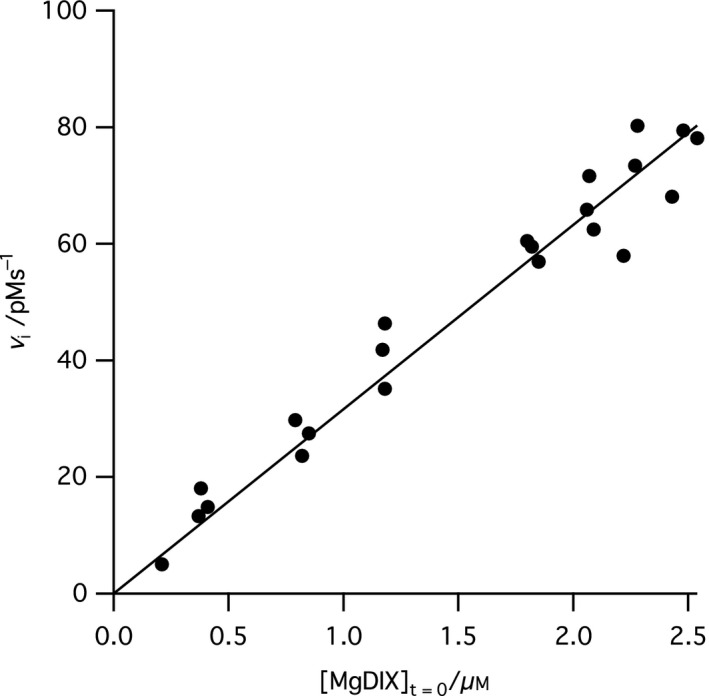
Initial rates of the uncatalyzed dechelation of Mg‐Deuteroporphyrin IX at 34 °C, 50 mm MOPS, 300 mm glycerol, *I* 0.1, pH 7.9, 1 mm DTT. The fitted line has a slope of 31.7 × 10^−6^ ± 0.9 × 10^−6^ s^−1^.

We have previously determined the equilibrium constant for magnesium chelation as 10^−6^; this observation was possible as MgD_IX_ will spontaneously lose Mg^2+^ over around 24 h 1. In the present work, we have determined the initial rates of this process (Fig. 4). In the concentration range examined, the process is first‐order with a rate constant of 31.7 × 10^−6^ ± 0.9 × 10^−6^ s^−1^. This implies that the second‐order rate constant for metal ion chelation is circa 30 × 10^−12^ m ^−1^·s^−1^.

Care was taken during all of these measurements to minimize illumination of the sample as MgD_IX_ is readily photolysed. Control experiments where MgD_IX_ was illuminated for the same length of time (1800 flashes) within a 5‐min time course showed negligible loss of porphyrin. We conclude that photolysis of MgD_IX_ is insignificant in these experiments.

## Discussion

Magnesium porphyrin formation is a slow and thermodynamically unfavorable process (Fig. 1). The enzyme magnesium chelatase catalyzes this reaction and uses the free energy of ATP hydrolysis to produce substantially more metalloporphyrin than would be expected from the *K*_eq_ alone. The effectiveness of magnesium chelatase at performing this reaction can be estimated in two distinct ways, firstly by rate acceleration when compared to the uncatalyzed reaction and secondly by the apparent shift in equilibrium position when compared to the uncoupled reaction. We show that magnesium chelatase not only provides a catalytic rate enhancement on the order of 30 × 10^15^ m ^−1^ but also generates at least 10^8^‐fold more Mg porphyrin than would be expected from the equilibrium constant alone.

We can compare the rate constants of catalyzed and uncatalyzed metal ion chelation to estimate how effectively magnesium chelatase accelerates metal ion chelation. Under our conditions, dechelation of MgD_IX_ is first‐order with a rate constant of (31.7 ± 0.9) × 10^−6^ s^−1^ (Fig. 4). Given the previous determination of *K*_eq_ as 10^−6^ 1, and if we assume that magnesium chelation can be described as a second‐order process, we can estimate the overall second‐order rate constant for metal ion chelation as circa 30 × 10^−12^ m ^−1^·s^−1^. Is this an unusually slow uncatalyzed reaction?

To answer this question we need rate information from a reference set of biologically relevant reactions; this is available for a set of first‐order reactions 13, 14. These reaction rate constants can be compared with the second‐order magnesium chelation process if we consider a biologically reasonable Mg^2+^ concentration of 1 mm (this varies *in vivo*, but is of the right order of magnitude). In this case, metal ion insertion will have a *t*_1/2_ of 0.7 million years, well within the range of half‐times seen for biologically important reactions in the absence of the appropriate catalyst 13, 14. As steady‐state rate constants for the enzyme‐catalyzed reaction are available 1 we can make a quantitative assessment of the rate enhancement.

The rate enhancement offered by magnesium chelatase (*k*_cat_/*k*_uncat_) is 400 × 10^6^ m or, in the presence of the activator protein Gun4, 550 × 10^6^ m 10. This is an estimate of the effectiveness of the active site environment in accelerating the reaction, but as metal ion chelation is not rate determining 15, *k*_cat_/*k*_uncat_ sets a lower limit for the rate enhancement.

Catalytic rate enhancement compares the effectiveness of catalyzed and uncatalyzed paths in converting substrate to product in solution. In single substrate and hydrolytic enzymes, (*k*_cat_/*K*_m_)/*k*_uncat_ is an effective measure of catalysis 13. In multi‐substrate, cooperative enzymes the situation is slightly more complex, but the kinetic parameter that best reflects the enzyme catalyzed reaction of free magnesium with porphyrin is $k_{\mathrm{cat}}/K_{m}^{\mathrm{DIX}}K_{0.5}^{\mathrm{Mg}}$. The ratio of this parameter to *k*_uncat_, 30 × 10^15^ m ^−1^, provides an estimate of the catalytic power of magnesium chelatase, with saturating ATP. We have shown previously that the biologically essential activator protein Gun4 exerts its major effect on the chelation reaction by a dramatic increase in $k_{\mathrm{cat}}/K_{m}^{\mathrm{DIX}}K_{0.5}^{\mathrm{Mg}}$ 10. As a result, the Gun4‐activated chelatase is a substantially more effective catalyst with a catalytic rate enhancement of at least 300 × 10^15^ m ^−1^.

Early kinetic studies of porphyrin metallation (reviewed in ref. 16) revealed a clear pattern in the rates of metal ion chelation by porphyrins; Zn^2+^ is inserted relatively rapidly and then the rate constants decrease in accordance with the Irving–Williams series. The transition metals react more quickly than Mg^2+^. These rates of metal ion insertion are correlated with the rate of inner‐sphere water exchange. But as the lifetime of bound water in the inner sphere of Mg^2+^ is roughly 1 μs 17, exchange is not likely to be rate determining in the magnesium chelatase reaction (1/*k*_cat_ = 75 s, ref. 1). Rather, both properties have a related cause; the small size and high charge density of the magnesium cation.

While mechanistic details of the magnesium chelation reaction are not known, parallels can be drawn with the well characterized ATP‐independent iron‐chelating enzyme ferrochelatase. The protoporphyrin IX ferrochelatase accelerates metal ion chelation by distorting the porphyrin ring of the substrate to present the pyrrole nitrogens to the incoming metal ion (reviewed in refs. 18, 19). This ring distortion and metal ion insertion are not the slowest steps in the reaction 20 and require no additional input of free energy from ATP hydrolysis—the free energy required to distort the ring is returned when the planar product is formed. The key difference between these two classes of chelatase comes in the relative preference of the metal ions for O vs. N type ligands; Fe(II) does not show a strong preference, while Mg(II) strongly prefers the more electronegative O over N. So if the gross mechanistic steps—distortion, deprotonation and metal ion donation—are similar in the two enzymes, the key difference is the relative energetics of the step where the metal exchanges O‐type ligands for N‐type: this exchange is significantly unfavorable in magnesium chelatase. This O‐for‐N exchange could be the final donation of metal ion into the porphyrin ring, or it could take place earlier if the metal ion is held in position by a set of nitrogen‐containing ligands. Understanding these steps requires structural information on the magnesium chelatase active site and experimental probes of the magnesium transfer reactions. The recent publication of a structure for the porphyrin‐binding domain of magnesium chelatase from *Synechocystis* suggests that information on the substrate‐binding sites of magnesium chelatase should soon become available 21.

Ultimately, the key problem in magnesium insertion into a porphyrin is that the reaction is energetically unfavorable; simply accelerating the rate of reaction is inadequate as the rate of the reverse reaction must also increase in proportion. As well as accelerating the reaction, magnesium chelatase can also generate a product: substrate ratio far from equilibrium through coupling metalloporphyrin formation to ATP hydrolysis.

The consequences of this coupling of ATP hydrolysis to metalloporphyrin formation can be clearly seen in reaction progress curves where coupled formation of reaction product is followed by uncoupled, uncatalyzed dechelation as the driving force is exhausted (Fig. 2). In the presence of ATP, magnesium chelatase produces a steady‐state product‐substrate ratio ([MgD_IX_]/[D_IX_][Mg^2+^]) shifted from the equilibrium constant by a factor of 10^8^, a free energy change of 53 kJ·mol^−1^. This substantial change is best treated as a lower limit because of the possibility of product inhibition or slight inactivation over these long reaction times. Using a reasonable *in vivo* ΔG°′ for ATP hydrolysis (−60 kJ·mol^−1^) 22 we may expect an ATP: metalloporphyrin stoichiometry of 1. In fact, stoichiometries of 15 are observed with the *Synechocystis* enzyme 1. Larger stoichiometries have been reported for chelatases from other species; the enzyme from *Rhodobacter capsulatus* requires 40 ATP to produce 1 metalloporphyrin 2.

One line of explanation proposes that the nucleotide hydrolysis is not solely used to provide thermodynamic driving force but is also used to accelerate the reaction 23. This explanation appears reasonable as perfectly efficient free energy transducing systems have a zero rate 24, 25. Magnesium chelatase, however, achieves a catalytic rate enhancement of the order of 10^17^ m ^−1^, a value not out of the usual range for enzyme‐catalyzed reactions. So it is not immediately obvious that magnesium chelatase needs to use nucleotide hydrolysis to provide rate acceleration.

Two other explanations appear reasonable. Firstly, in addition to providing free energy the ATPase could also regulate the chelatase, by controlling the conversion of active and inactive forms of the enzyme in a manner analogous to many GTPases 26, 27. Alternatively, the *in vitro* estimates of the ATPase stoichiometry could be overestimates of the *in vivo* situation; perhaps through the presence of an additional coupling factor. Magnesium chelatase is activated by further protein–protein interactions, most notably with Gun4, but this interaction does not substantially increase the amount of metalloporphyrin formed in our *in vitro* assays. The amount of Mg porphyrin formed by the magnesium chelatase from *R. capsulatus*, is substantially increased by the next enzyme in the pathway, BchM, but the ATPase stoichiometry under these conditions is not known 28.

As the ATP stoichiometry is high (15–40; refs. 1, 2) and the number of ATPase active sites is low (6 described in ref. 29) we suggest that the link between ATP hydrolysis and metal ion chelation is probabilistic. That is the ATP hydrolysis cycle transiently shifts the ChlH subunit into a chelation competent conformation that has a well defined probability—1/15 in the *Synechocystis* enzyme—of inserting a magnesium. This suggests that only 6–7% of these transiently activated chelatase subunits successfully insert magnesium before the ATPase cycle rolls on and the AAA^+^ ring resets for another attempt.

Magnesium chelatase uses the free energy of ATP hydrolysis to offer a substantial thermodynamic benefit—the enzyme can hold the magnesium chelation reaction 53 kJ·mol^−1^ from equilibrium. In general, the AAA^+^ ATPases use the free energy of ATP hydrolysis to drive macromolecular remodeling reactions, often with a high ATP stoichiometry 1. To the best of our knowledge, an analysis of the sort presented here has not been carried out on any other member of the AAA^+^ superfamily. If magnesium chelatase is representative, then our work suggests that the AAA^+^ motor unit can drive coupled reactions a factor of 10^8^ from their equilibrium positions.

## Author contributions

NBPA conducted the majority of experiments. AAB produced the Gun4 protein. NBPA, CNH, and JDR wrote the paper.
